# Postoperative Quadriceps Symmetry, Knee Laxity, and Single-Leg Hop Performance in Association with 24-Month Ipsilateral Graft Retear After Anterior Cruciate Ligament Reconstruction: A Cohort Study

**DOI:** 10.3390/life16091546

**Published:** 2026-09-16

**Authors:** Georgios Kakavas, Nikolaos Malliaropoulos, Florian Forelli

**Affiliations:** 1 Fysiotek Spine & Sports Lab, 17562 Athens, Greece; 2 Thessaloniki Sports Medicine Clinic, 54622 Thessaloniki, Greece; 3 Sports Clinic, Rheumatology Department, Barts Health NHS Trust, London E1 1BB, UK; 4 Haute-Ecole Arc Santé, HES-SO University of Applied Sciences and Arts Western Switzerland, 2800 Delémont, Switzerland; 5 Orthopaedic Surgery Department, Clinic of Domont, Ramsay Healthcare, OrthoLab, 95330 Domont, France; 6 Société Française des Masseurs-Kinésithérapeutes du Sport Lab, 93380 Pierrefitte-Sur-Seine, France

**Keywords:** anterior cruciate ligament reconstruction, graft retear, GNRB, knee laxity, quadriceps strength, limb symmetry index, single-leg hop

## Abstract

Background: Ipsilateral graft retear after anterior cruciate ligament reconstruction (ACLR) remains a major concern in athletes, while the relationship between postoperative mechanical and functional measures and retear remains uncertain. Methods: This cohort study included 48 athletes after primary unilateral ACLR with hamstring-tendon autograft. Follow-up was scheduled at 6, 9, and 12 months (184 ± 13, 276 ± 17, and 368 ± 22 days), and the analytic export retained one postoperative value per athlete for GNRB side-to-side laxity at 250 N, concentric isokinetic quadriceps limb symmetry index (LSI) at 60°/s, single-leg hop-for-distance LSI, and patient-reported outcomes, without preserving the originating visit. MRI-confirmed ipsilateral graft retear was recorded within 24 months after clinically suspected reinjury; retear dates were not retained. Results: Fourteen athletes (29.2%) sustained a retear. Quadriceps LSI was lower in the retear group than in the no-retear group (81.3 ± 7.5% vs. 88.1 ± 9.0%; *p* = 0.011), and each 1-SD higher quadriceps LSI was associated with lower retear odds (OR 0.43, 95% CI 0.21–0.89). In an exploratory threshold analysis, quadriceps LSI < 85% was associated with a higher retear proportion (RR 3.21, 95% CI 1.17–8.82). Associations for knee laxity, hop performance, and patient-reported outcomes were not statistically robust. Conclusions: Postoperative quadriceps symmetry showed the clearest association with 24-month graft-retear status. However, because the originating measurement visit and retear dates were unavailable, temporal precedence could not be established, and the exploratory <85% LSI finding should not be interpreted as a validated return-to-sport threshold.

## 1. Introduction

Anterior cruciate ligament (ACL) reconstruction is intended to restore knee stability and enable return to demanding activity, yet ipsilateral graft retear remains a major concern, particularly in young and highly active athletes. Graft failure can prolong rehabilitation, delay or prevent return to previous sport, and expose the knee to further surgical intervention. Young athletes returning to pivoting sport may experience second-ACL-injury rates exceeding 20% when ipsilateral graft rupture and contralateral ACL injury are considered together, and prospective cohorts confirm substantially greater 24-month second-injury incidence after ACL reconstruction than in uninjured athletes [1,2]. Younger age, high activity level, and selected graft-related factors are established determinants of subsequent graft failure [3,4], while systematic evidence confirms that reinjury risk persists beyond the immediate postoperative period [5,6]. These observations have moved return-to-sport (RTS) decision-making away from chronology alone toward risk management incorporating biological recovery, knee function, psychological readiness, and sport exposure. Emerging concepts of ACL tissue healing further reinforce that biological status cannot be inferred from postoperative time alone [7].

Contemporary consensus statements and rehabilitation guidelines therefore recommend criterion-based progression [8,9,10,11]. Persistent deficits in strength, balance, and movement quality have been documented after ACLR [12], and individualized loading approaches, including velocity-based resistance training, emphasize restoration of force, power, and neuromuscular capacity in mid- and late-stage rehabilitation [13]. Neuromuscular alterations may also extend beyond the quadriceps; gastrocnemius activation deficits and altered running biomechanics have been reported after ACLR [14]. Rehabilitation recovery is non-linear and should be progressed according to biological, neuromuscular, psychological, and sport-specific constraints [8,9,10,11]. Nevertheless, the prognostic value of commonly used RTS tests remains uncertain. Greater quadriceps symmetry and delayed RTS were associated with lower reinjury rates in the Delaware-Oslo cohort [15]. Separately, failure to meet a six-item discharge battery was associated with higher graft-rupture risk in a cohort of professional athletes [16]. Conversely, recent systematic reviews show inconsistent associations between isolated strength or hop measures and subsequent ACL injury [17,18], and substantial heterogeneity persists in RTS batteries and testing timelines [19,20]. This uncertainty is clinically important because LSI-based criteria are widely used to support clearance decisions despite substantial variation in test selection, scoring, and threshold definition. Thus, familiar LSI thresholds should not be interpreted as validated stand-alone safety cut-offs.

Mechanical stability represents another potentially informative domain. Instrumented arthrometry provides an objective estimate of anterior tibial translation, and registry studies have reported greater revision hazards among patients with increased postoperative laxity [21,22]. However, associations depend on population, device, loading protocol, timing, and outcome definition. The GNRB arthrometer was developed to standardize anterior tibial translation measurement [23], but values obtained with different laximeters are not interchangeable [24]. Previous work also suggests that progressive quadriceps strengthening can improve recovery without necessarily increasing measured graft laxity [25,26], supporting simultaneous surveillance of mechanical and neuromuscular status. Whether such mechanical measures add clinically meaningful prognostic information beyond strength and functional testing remains unresolved.

Patient-reported outcomes add information not captured by performance testing. IKDC, KOOS, and ACL-RSI quantify perceived function and psychological readiness, and ACL-RSI has been associated with second injury in selected younger cohorts [27,28]. Limb symmetry indices are also clinically convenient but may overestimate recovery when the contralateral limb is deconditioned [29]. Ecological RTS frameworks therefore advocate integrating quantitative tests with movement quality, sport-specific exposure, and contextual information [30]. This integrated question is particularly relevant for high-activity athletes returning to pivoting sports. Yet relatively few clinical cohorts have examined laxity, quadriceps symmetry, hop performance, and patient-reported outcomes together against clinically ascertained, MRI-confirmed ipsilateral graft retear within 24 months. We therefore examined their associations with 24-month retear status after primary ACLR and performed an exploratory assessment of a parsimonious multivariable model. We hypothesized that poorer quadriceps and hop symmetry and greater postoperative laxity would be associated with retear status.

## 2. Materials and Methods

### 2.1. Study Design, Setting, and Reporting

This observational cohort study was conducted at Fysiotek Sports Lab, Athens, Greece, during the 2023–2025 study period. Athletes undergoing primary ACL reconstruction (ACLR) entered a standardized postoperative follow-up pathway with scheduled assessments and clinical surveillance for suspected ipsilateral graft retear over a 24-month postoperative horizon. The present analysis examined the association between postoperative mechanical, strength, functional, and patient-reported measures and the binary 24-month graft-retear outcome. Reporting was structured according to the STROBE statement for cohort studies [31]. Because an exploratory multivariable model was evaluated, relevant transparency principles from TRIPOD+AI were also applied [32].

### 2.2. Ethics and Consent

The study was conducted in accordance with the Declaration of Helsinki. Fysiotek Sports Lab served as the clinical assessment site, while ethical review and approval were provided by the University of Athens, Department of Physical Education and Sports Science Ethics Board on 1 October 2022. Informed consent was obtained from all participants involved in the study.

### 2.3. Participants and Cohort Assembly

Eligibility criteria were age 16–45 years, MRI-confirmed primary unilateral ACL rupture, participation in pivoting or contact sport before injury, high preinjury activity operationally defined for this study as Tegner activity level ≥ 7 and Marx Activity Rating Scale ≥ 12, ACLR with hamstring-tendon autograft, and ability to adhere to the standardized rehabilitation and follow-up pathway. Exclusion criteria were previous ACL injury or reconstruction in either knee; multiligament injury requiring reconstruction of the posterior cruciate ligament, grade III medial collateral ligament, lateral collateral ligament, or posterolateral corner; complex peri-knee fracture; severe cartilage defects requiring advanced cartilage procedures; concomitant meniscal surgical procedures; and neurological or systemic conditions affecting gait or strength.

Forty-eight consecutive eligible athletes constituted the analytic cohort. Complete data were available for the candidate variables analyzed, and 24-month graft-retear status was available for all 48 athletes. Consecutive inclusion, a standardized postoperative assessment pathway, restriction to isolated primary ACLR with hamstring-tendon autograft, and MRI-based confirmation of clinically suspected graft retear were used to reduce avoidable selection and outcome-misclassification bias. No a priori sample-size calculation was performed; study size was determined by the consecutive eligible athletes with complete outcome data available in the study database.

### 2.4. Surgery and Rehabilitation Pathway

All ACL reconstructions were performed using an anatomic single-bundle technique with hamstring-tendon autograft. Femoral tunnel placement used an anteromedial portal or outside-in approach, and tibial tunnel placement targeted the native ACL insertion. Graft diameter, fixation method, surgeon identifier or number, and the distribution of femoral tunnel drilling approaches were not retained in the analytic dataset and therefore could not be examined. To reduce surgical heterogeneity in the analytic cohort, athletes undergoing concomitant meniscal surgical procedures were excluded, thereby restricting the analysis to isolated primary ACLR.

Postoperative rehabilitation followed a phase-based pathway. During weeks 0–6, management emphasized symptom control, restoration of range of motion, quadriceps activation, and progressive weight bearing. During weeks 6–12, rehabilitation progressed to strengthening, cycling, and proprioceptive training. From approximately 3–6 months, running, plyometric exercise, neuromuscular drills, and sport-specific agility were introduced. From approximately 6 months onward, advanced sport drills and objective return-to-sport (RTS) testing were incorporated. Contact training and competition were not permitted before 9 months. The specific objective clearance criteria applied to individual athletes were not retained in the analytic dataset. Individual rehabilitation exposure, detailed session-level dose, and actual RTS dates were also not retained; consequently, these variables were not modeled as exposures or covariates.

### 2.5. Postoperative Assessment Schedule and Analytic Data Structure

Participants underwent standardized postoperative assessments at three predefined follow-up points: 6 months (184 ± 13 days), 9 months (276 ± 17 days), and 12 months (368 ± 22 days) after ACLR. The assessment pathway included instrumented knee laxity, isokinetic quadriceps strength, single-leg hop performance, and patient-reported outcomes. For the present retear analysis, however, the available analytic export retained one postoperative value per athlete for each outcome but did not preserve the originating visit, the rule used to select the retained value when more than one scheduled assessment was available, or whether all retained measures for a given athlete came from the same visit. These features could not be reconstructed from the source records. The analysis therefore used the verified exported values, did not model within-participant trajectories across the three scheduled visits, and could not determine whether each retained measurement preceded graft retear. Accordingly, the findings should be interpreted as postoperative associations with 24-month retear status rather than as visit-specific or temporally validated predictions. Procedural details not preserved in the source records were not retrospectively reconstructed.

#### 2.5.1. Instrumented Knee Laxity

Anterior tibial translation was assessed bilaterally with a GNRB^®^ arthrometer (GENOUROB SAS, Laval, France) with the participant supine and the knee positioned at approximately 20–30° of flexion. The source records did not preserve whether an identical fixed flexion angle within this range was used for every participant, and no more precise angle specification was retrospectively reconstructed. Testing was performed at a standardized anterior load of 250 N. Three trials were obtained and the mean value was retained for each knee. The prespecified arthrometric variable for analysis was the side-to-side difference (SSD) in anterior tibial translation, expressed in millimetres and calculated as reconstructed-knee translation relative to the contralateral knee. A fixed force level was used because GNRB measurements are load dependent and device specific [23,24].

#### 2.5.2. Isokinetic Quadriceps Strength

Quadriceps strength was assessed bilaterally using isokinetic dynamometry in concentric mode at an angular velocity of 60°/s. Peak torque was retained for each limb. The source records did not preserve the number of repetitions per limb or whether the retained peak torque represented the best repetition or another summary, and these procedural details were not retrospectively reconstructed. Quadriceps limb symmetry index (LSI) was calculated as reconstructed-limb peak torque divided by contralateral-limb peak torque × 100. The LSI was used as a relative measure of postoperative quadriceps recovery; it was not treated as equivalent to recovery of absolute strength because the contralateral limb may itself change after ACLR [29].

#### 2.5.3. Single-Leg Hop for Distance

Functional performance was assessed with the single-leg hop for distance. Each athlete performed three trials per limb. The greatest valid distance was retained for the reconstructed and contralateral limbs. The operational criteria used in the source testing record to designate a trial as invalid were not preserved in the analytic dataset and were not retrospectively reconstructed. Hop LSI was calculated as best reconstructed-limb distance divided by best contralateral-limb distance × 100. Hop LSI was interpreted as a performance-symmetry measure rather than as a direct measure of movement quality.

#### 2.5.4. Patient-Reported Outcomes

The analytic dataset included the International Knee Documentation Committee (IKDC) subjective score, the Knee injury and Osteoarthritis Outcome Score Sport and Recreation subscale (KOOS-Sport), and the Anterior Cruciate Ligament–Return to Sport after Injury (ACL-RSI) score. Higher values indicate better self-reported knee function, better sport/recreation function, and greater psychological readiness to return to sport, respectively. The specific language versions administered in this Greek cohort were not preserved in the analytic source records; therefore, version-specific validation references could not be reconstructed retrospectively. These outcomes were analyzed as continuous variables and were considered complementary to the mechanical and performance-based measures.

### 2.6. Primary Outcome and Retear Ascertainment

The primary outcome was ipsilateral ACL graft retear status within 24 months after the index ACLR. Retear status was available for all 48 athletes. However, the analytic dataset did not document attendance at a dedicated 24-month clinical visit for every participant; therefore, availability of 24-month retear status should not be interpreted as confirmation that all athletes completed a formal 24-month assessment. Retear ascertainment was event-triggered: clinically suspected reinjury prompted magnetic resonance imaging, and all 14 recorded retear events were MRI-confirmed graft disruptions. The analytic dataset did not document systematic scheduled MRI screening of asymptomatic athletes. Event dates were not retained in the study database; consequently, the outcome was analyzed as a binary 24-month event rather than with time-to-event methods. Because the originating visit of each exported postoperative measure was also unavailable, temporal precedence between the retained measurement and graft retear could not be verified; the study therefore estimates postoperative associations with retear status rather than validated prospective prediction from a specific postoperative time point.

### 2.7. Study Size, Missing Data, and Model Complexity

The analytic sample comprised all 48 consecutive eligible athletes with complete values for the variables included in the present analysis and complete 24-month retear ascertainment. Fourteen retear events were observed. No a priori sample-size calculation had been performed. A post hoc contextual check using the observed retear proportion (14/48 = 0.292) showed that the Riley criterion for estimating the overall outcome proportion with a ±0.05 margin of error alone would require 318 participants [33]. Because the full Riley calculation additionally requires an anticipated Cox-Snell R^2^ specified independently of the development data, which was unavailable for this exploratory analysis, no unique full minimum sample size was calculated. Accordingly, the 318-participant figure should be interpreted only as a lower-bound contextual criterion from the outcome-proportion component of the Riley framework, not as the full Riley minimum sample size. The available cohort therefore does not meet sample-size requirements for formal prediction-model development. Given the small event count, the multivariable analysis was deliberately restricted, penalized, and treated as exploratory, with internal bootstrap procedures used to quantify optimism. No imputation was required for the analyzed variables because the analytic dataset was complete for the variables retained in the models.

### 2.8. Statistical Analysis

Continuous variables were summarized as mean ± standard deviation (SD), with medians and ranges used during data verification. Formal normality-test results were not retained; medians and ranges were reviewed during data verification to assess distributional plausibility. Categorical variables were summarized as counts and percentages. Retear and no-retear groups were compared using Welch two-sample tests for continuous variables, with mean differences, 95% confidence intervals (CIs), and Cohen’s d effect sizes; sex distribution was examined with Fisher exact testing. Primary association analyses used separate univariable logistic regression models. Continuous predictors were standardized to one sample SD so that odds ratios (ORs) represent the change in retear odds per 1-SD higher value. Sex was coded as male versus female.

Threshold analyses were secondary and exploratory. Prespecified exploratory cut-offs were quadriceps LSI < 85%, hop-for-distance LSI < 90%, SSD ≥ 3 mm, and SSD ≥ 5 mm. For each threshold, retear proportions, risk ratios (RRs), 95% CIs, and Fisher exact *p* values were calculated. These cut-offs were used only to describe cohort-specific patterns and were not treated as validated clinical decision thresholds or independent return-to-sport criteria.

For multivariable exploration, a four-variable L2-penalized logistic regression included SSD, quadriceps LSI, hop-for-distance LSI, and sex. Continuous variables were standardized, and regularization strength was fixed at C = 1 without data-driven tuning to avoid an additional optimization step in a dataset with only 14 events. Predictor uncertainty was summarized with percentile intervals from 2000 bootstrap resamples. Apparent discrimination was quantified using the area under the receiver operating characteristic curve (AUC), and bootstrap optimism was estimated by comparing performance in each bootstrap sample with performance of the bootstrap-fitted model in the original sample. The apparent Brier score was also calculated. An unpenalized logistic model using the same four predictors was examined as a sensitivity analysis. No automated variable selection was performed.

All tests were two sided. Because the study was exploratory and multiple correlated outcomes were examined, *p* values were interpreted alongside effect sizes and CIs rather than as isolated dichotomous evidence of association; no multiplicity correction was applied. Cohen’s d for Table 1 was calculated as the retear-minus-no-retear mean difference divided by the pooled SD. Analyses were reproduced in Python 3.13.5 using SciPy 1.17.0, statsmodels 0.14.6, and scikit-learn 1.8.0.

## 3. Results

### 3.1. Cohort and Retear Incidence

The analytic cohort comprised 48 athletes, including 32 women (66.7%) and 16 men (33.3%), with a mean age of 25.6 ± 4.5 years (range 17–32). All participants underwent isolated primary ACLR with hamstring-tendon autograft; concomitant meniscal surgical procedures were excluded. All participants met the study-defined high-activity eligibility thresholds (Tegner activity level ≥ 7; Marx Activity Rating Scale ≥ 12). Fourteen athletes sustained an ipsilateral graft retear within 24 months, corresponding to a cumulative proportion of 29.2% (95% CI 18.2–43.2%). Retear occurred in 10/32 women (31.3%) and 4/16 men (25.0%); this difference was not statistically supported (Fisher *p* = 0.746).

### 3.2. Postoperative Measures by Retear Status

Quadriceps LSI was lower in the retear group than in the no-retear group (81.3 ± 7.5% vs. 88.1 ± 9.0%), with a mean difference of −6.87 percentage points (95% CI −12.05 to −1.69; *p* = 0.011). Hop-test LSI was also lower on average in the retear group, but the difference was imprecise and included the null (−3.89 percentage points, 95% CI −9.15 to 1.36; *p* = 0.140). Mean SSD was 3.98 ± 1.66 mm in the retear group and 3.67 ± 1.17 mm in the no-retear group (mean difference +0.31 mm, 95% CI −0.71 to 1.33; *p* = 0.537). No statistically robust between-group differences were observed for IKDC, KOOS-Sport, ACL-RSI, or age (Table 1).

### 3.3. Univariable Associations

In standardized univariable logistic models, quadriceps LSI showed the clearest association with graft retear: each 1-SD higher quadriceps LSI was associated with lower retear odds (OR 0.43, 95% CI 0.21–0.89; *p* = 0.022). Associations for SSD (OR 1.27, 95% CI 0.67–2.42), hop-for-distance LSI (OR 0.62, 95% CI 0.33–1.19), age, IKDC, KOOS-Sport, ACL-RSI, and sex all had CIs crossing the null (Table 2).

### 3.4. Secondary Threshold Analyses

Retear occurred in 10/21 athletes (47.6%) with quadriceps LSI < 85% versus 4/27 (14.8%) with quadriceps LSI ≥ 85% (RR 3.21, 95% CI 1.17–8.82; Fisher *p* = 0.024). For hop-for-distance LSI, retear occurred in 11/28 athletes (39.3%) below 90% versus 3/20 (15.0%) at or above 90% (RR 2.62, 95% CI 0.84–8.19; *p* = 0.108). SSD thresholds did not show a monotonic risk gradient: retear risk was 26.5% for SSD ≥ 3 mm versus 35.7% for SSD < 3 mm, while risk was 41.7% for SSD ≥ 5 mm versus 25.0% for SSD < 5 mm (Table 3).

### 3.5. Exploratory Penalized Multivariable Model

In the L2-penalized model, shrinkage-adjusted ORs were 1.56 per 1-SD higher SSD, 0.41 per 1-SD higher quadriceps LSI, 0.55 per 1-SD higher hop-for-distance LSI, and 0.65 for male versus female sex. Bootstrap percentile intervals were wide for SSD and sex and excluded 1.0 for quadriceps LSI and, narrowly, hop-for-distance LSI (Table 4). Because penalization changes the inferential target and the event count was small, these estimates should not be labelled independent causal predictors.

The apparent AUC was 0.842. Mean bootstrap optimism was 0.065, yielding an optimism-corrected AUC estimate of 0.778. The apparent Brier score was 0.164 and the optimism-corrected estimate was 0.199. Because no external dataset was available and only 14 events occurred, these values describe exploratory internal performance only. In an unpenalized sensitivity model using the same predictors, the ORs were 2.01 for SSD (95% CI 0.82–4.95; *p* = 0.127), 0.32 for quadriceps LSI (95% CI 0.13–0.78; *p* = 0.013), 0.47 for hop-for-distance LSI (95% CI 0.22–1.02; *p* = 0.056), and 0.36 for male sex (95% CI 0.05–2.57; *p* = 0.307). The shift from the univariable quadriceps estimate (OR 0.43) to the more extreme unpenalized multivariable estimate (OR 0.32), followed by attenuation with L2 penalization (OR 0.41), was interpreted as a combination of covariate adjustment and sparse-event model instability rather than evidence of a stronger independent effect.

## 4. Discussion

The principal finding was that quadriceps limb symmetry showed the clearest association with ipsilateral graft retear within 24 months, whereas GNRB side-to-side laxity, single-leg hop-for-distance symmetry, IKDC, KOOS-Sport, and ACL-RSI did not show statistically robust univariable associations and their confidence intervals were generally wide. Athletes with retear had a quadriceps LSI 6.9 percentage points lower than those without retear, and the observed retear proportion was higher when quadriceps LSI was <85%; this threshold comparison was exploratory and cohort-specific. In the exploratory penalized model, greater quadriceps symmetry remained associated with lower retear odds. The apparent AUC was 0.84 and decreased to approximately 0.78 after bootstrap optimism correction; with only 14 events, this represents exploratory internal performance rather than validated discrimination. The 29.2% 24-month retear proportion is high and should be interpreted in the context of this selected high-activity pivoting/contact-sport cohort and the wide 95% CI (18.2–43.2%) produced by the small sample; the available data do not permit attribution of the elevated rate to a specific exposure.

The quadriceps finding is biologically and functionally plausible because quadriceps capacity contributes to force absorption, deceleration, landing, and restoration of knee loading after ACLR. However, biological plausibility should not be equated with consistent prognostic evidence, because systematic reviews report inconsistent associations between isolated strength measures and graft rupture [17,18,34,35]. Greater quadriceps symmetry before RTS has previously been associated with lower reinjury rates [15], while persistent strength and movement-quality deficits have been documented in football players after ACLR [12]. Early open kinetic chain strengthening can improve quadriceps recovery without measurable increases in graft laxity [25,26], and this is consistent with contemporary criterion-based rehabilitation guidance [10,11]. Velocity-based resistance training may further individualize loading and target strength, power, and rapid force production during later rehabilitation [13]. Persistent neuromuscular inhibition may help explain why apparently satisfactory global function can coexist with incomplete recovery [11]. In addition, LSI can overestimate recovery when the contralateral limb is deconditioned [29], and a universal LSI threshold is not supported. The <85% finding should therefore be considered cohort-specific and should not be used as an independent return-to-sport criterion.

Hop-for-distance LSI showed a directionally consistent but imprecise association: retear occurred in 39.3% of athletes with LSI < 90% versus 15.0% at or above 90%, but the confidence interval included the null. This agrees with evidence that multidomain RTS batteries may be informative [15,16,17], whereas isolated hop tests often have limited prognostic value [18,35]. Dual-task testing indicates that hop distance may not fully capture persistent movement-quality deficits [36], and lower-limb neuromuscular alterations during running, including gastrocnemius activation deficits, further show that satisfactory performance on a single task does not ensure restoration across sporting demands [14]. Distance symmetry can also conceal altered knee loading or landing strategies. Hop results should therefore be interpreted alongside strength, movement quality, symptoms, psychological readiness, and sport exposure [8,9,10,11,30].

Contrary to our hypothesis, GNRB SSD at 250 N was not clearly associated with retear. The between-group difference was only 0.31 mm, and neither the ≥3-mm nor ≥5-mm threshold demonstrated a supported risk increase. This differs from registry studies linking greater postoperative arthrometric laxity with revision risk [21,22]. Our smaller cohort used MRI-confirmed graft retear rather than revision surgery as the outcome, and GNRB measurements are device- and load-dependent [23,24]. The result therefore indicates absence of a demonstrable laxity signal in this sample, not that postoperative laxity is clinically irrelevant. IKDC, KOOS-Sport, and ACL-RSI were likewise not associated with retear, although these measures remain important for domains not captured by strength or hop testing [27,28].

Several limitations constrain inference. Only 14 retears occurred, limiting model complexity and precision; no a priori sample-size calculation was performed, and the available cohort does not meet formal sample-size requirements for prediction-model development [32,33]. Penalization and bootstrap validation reduced but did not remove overfitting concerns. Restriction to isolated primary ACLR with hamstring-tendon autograft reduced surgical heterogeneity but limits generalizability. Graft diameter, fixation method, surgeon variability, and the distribution of femoral tunnel drilling approaches were unavailable; surgical and graft-related factors may influence failure risk [4,37]. Rehabilitation exposure, exact RTS date, and sport-exposure volume were also unavailable and may influence reinjury [34,38,39,40]. Although follow-up visits were standardized at 6, 9, and 12 months, the analytic export did not preserve the originating visit, the rule used to select the retained value, or whether all measures for an athlete came from the same visit; retear event dates were also unavailable. Consequently, temporal precedence could not be verified, and differential measurement timing between athletes with and without retear is possible. This potential measurement-timing bias could contribute to the observed quadriceps association and precludes interpreting low quadriceps LSI as a demonstrated risk factor rather than a postoperative correlate. Retear ascertainment in the analytic dataset was based on clinical suspicion followed by MRI, and systematic scheduled MRI screening of asymptomatic athletes was not documented; asymptomatic partial or subclinical graft compromise therefore cannot be excluded, creating possible verification and outcome-misclassification bias. Although 24-month retear status was available for all athletes, attendance at a dedicated 24-month clinical visit was not documented for every participant. The cohort was predominantly female (66.7%), and the small sample did not support sex-stratified inference; generalizability to cohorts with different sex distributions is therefore uncertain. Additional procedural details requested in this review—including exact GNRB flexion-angle standardization, isokinetic repetition and peak-torque selection rules, hop invalid-trial criteria, and the language versions of patient-reported outcomes—were not preserved in the source records and could not be reconstructed retrospectively. Future multicenter studies should retain visit-level, surgical, procedural, and sport-exposure data, use time-to-event methods, incorporate systematic outcome surveillance where feasible, and externally validate any prognostic model. Clinically, these findings support multidimensional RTS assessment with particular attention to quadriceps recovery while avoiding overinterpretation of any single threshold.

## 5. Conclusions

Within this exploratory 48-athlete cohort, postoperative quadriceps limb symmetry showed the clearest association with 24-month ipsilateral ACL graft-retear status, but temporal precedence between the retained measurement and retear could not be established. Single-leg hop-for-distance asymmetry showed a clinically relevant but statistically imprecise pattern, while neither continuous GNRB SSD measured at 250 N nor the prespecified SSD thresholds showed a clear association with retear. Patient-reported outcomes also showed no statistically robust association with retear in this sample. These findings support multidimensional RTS assessment with particular attention to quadriceps recovery, but the exploratory <85% LSI finding is cohort-specific and does not establish a validated return-to-sport threshold or stand-alone clearance criterion.

## Figures and Tables

**Table 1:** Postoperative characteristics according to 24-month graft-retear status.

Variable	No Retear (*n* = 34)	Retear (*n* = 14)	Mean Difference (95% CI)	Cohen’s d	*p* Value
Age, years	25.3 ± 4.7	26.1 ± 4.1	+0.82 (−1.97 to 3.61)	0.18	0.552
SSD laxity, mm	3.67 ± 1.17	3.98 ± 1.66	+0.31 (−0.71 to 1.33)	0.23	0.537
Quadriceps LSI, %	88.1 ± 9.0	81.3 ± 7.5	−6.87 (−12.05 to −1.69)	−0.79	0.011
Hop-test LSI, %	87.9 ± 8.6	84.0 ± 7.8	−3.89 (−9.15 to 1.36)	−0.47	0.140
IKDC score	76.4 ± 15.7	73.5 ± 16.9	−2.84 (−13.73 to 8.05)	−0.18	0.595
KOOS-Sport	70.6 ± 17.9	69.9 ± 14.6	−0.69 (−10.81 to 9.43)	−0.04	0.890
ACL-RSI	68.4 ± 22.3	64.2 ± 24.2	−4.22 (−19.77 to 11.33)	−0.18	0.580

Note: Difference is retear minus no retear. Cohen’s d is signed in the same direction (retear minus no retear). Continuous-variable *p* values are from Welch tests. Values are mean ± SD. CI, confidence interval; LSI, limb symmetry index; SSD, side-to-side difference.

**Table 2:** Univariable logistic associations with 24-month graft retear.

Predictor	OR	95% CI	*p* Value
Age	1.21	0.63–2.30	0.565
SSD laxity	1.27	0.67–2.42	0.464
Quadriceps LSI	0.43	0.21–0.89	0.022
Hop-test LSI	0.62	0.33–1.19	0.152
IKDC	0.83	0.44–1.57	0.572
KOOS-Sport	0.96	0.51–1.80	0.896
ACL-RSI	0.83	0.44–1.56	0.555
Male sex vs. female	0.73	0.19–2.85	0.654

Note: ORs for continuous variables are per 1-SD higher value; the sex OR compares male with female. CI, confidence interval; OR, odds ratio; SD, standard deviation.

**Table 3:** Exploratory threshold analyses.

Threshold	Risk If Threshold Met	Risk If Not Met	RR	95% CI	Fisher *p*
Quadriceps LSI < 85%	10/21 (47.6%)	4/27 (14.8%)	3.21	1.17–8.82	0.024
Hop-test LSI < 90%	11/28 (39.3%)	3/20 (15.0%)	2.62	0.84–8.19	0.108
SSD ≥ 3 mm	9/34 (26.5%)	5/14 (35.7%)	0.74	0.30–1.82	0.728
SSD ≥ 5 mm	5/12 (41.7%)	9/36 (25.0%)	1.67	0.69–4.00	0.294

Note: Threshold analyses were prespecified as exploratory and should not be interpreted as validated clinical decision rules. CI, confidence interval; RR, risk ratio.

**Table 4:** Exploratory L2-penalized logistic model.

Predictor	Penalized OR	Bootstrap 95% Interval
SSD laxity, per 1 SD	1.56	0.76–3.60
Quadriceps LSI, per 1 SD	0.41	0.20–0.78
Hop-test LSI, per 1 SD	0.55	0.26–0.96
Male sex vs. female	0.65	0.26–1.59

Note: Continuous predictors are expressed per 1-SD higher value. Intervals are percentile intervals from 2000 bootstrap resamples. OR, odds ratio; SD, standard deviation; SSD, side-to-side difference.

## Data Availability

The de-identified data that support the findings of this study are available from the corresponding author upon reasonable request.
